# Cholesteryl-Modification of a Glucomannan from *Bletilla striata* and Its Hydrogel Properties

**DOI:** 10.3390/molecules19079089

**Published:** 2014-06-30

**Authors:** Mengshan Zhang, Lin Sun, Wencui Zhao, Xiaoxia Peng, Fuqiang Liu, Yanping Wang, Yajing Bi, Hengbi Zhang, Yifa Zhou

**Affiliations:** 1 School of Life Sciences, Northeast Normal University, Changchun 130024, China; E-Mails: zhangms253@nenu.edu.cn (M.Z.); sunl925@nenu.edu.cn (L.S.); pengxx039@nenu.edu.cn (X.P.); 2 Pharmacy Department, Chinese People’s Liberation Army 208 Hospital, Changchun 130062, China; E-Mails: zhaowencui208@126.com (W.Z.); cclfq@126.com (F.L.); aswangyanping@163.com (Y.W.); asbiyajing@163.com (Y.B.); zqi8229@126.com (H.Z.)

**Keywords:** cholesteryl-modification, glucomannan, *Bletilla striata*, hydrogel property

## Abstract

A glucomannan-type polysaccharide, named BSP, was obtained from the tubers of *Bletilla striata* by ultrasonic-assisted extraction, ethanol precipitation, deproteination and gel-permeation chromatography. HPLC analysis revealed that BSP contained mannose and glucose in the molar ratio of 3.5:1. Its molecular weight (Mw) was estimated to be 20 kDa. Methylation analysis, FT-IR and NMR analyses indicated that BSP consisted of (1→4)-linked β-d-glucopyranosyl residues and (1→4)-linked β-d-mannopyranosyl residues. Cholesteryl succinate was linked to BSP to make it more amphiphilic and the degree of substitution of cholesteryl succinate-BSP was 3.2%. The critical micelle concentration of modified BSP was 0.001 mg/mL, suggesting it could self-assemble into nanoparticles in aqueous solution.

## 1. Introduction

*Bletilla striata (Thunb.) Reichb. f*. is a perennial herbaceous plant, used as a traditional medicine for thousands of years in East Asian countries [1]. *Bletilla striata* polysaccharide (BSP) gel is a natural hydrogel used for preparing biological adhesives [2]. BSP microspheres have been prepared for directed targeting, drug delivery, controlled release and for their anti-tumor activities [3]. Glucomannan with a backbone of (1→4)-linked β-d-mannose and glucose in general is the dominant neutral polysaccharide in *Bletilla striata* [4,5]. A recent study reported a new glucomannan obtained by hot water extraction from *Bletilla striata* and subsequent purification by ion-exchange chromatography that was mainly composed of (1→2)-linked α-d-mannopyranose and (1→4)-linked β-d-glucopyranose residues [6]. It is also reported that the tubers of *Bletilla striata* contained abundant glucomannan, responsible for its bioactivities [7]. Although polysaccharides from *Bletilla striata* possess various pharmacological functions, few studies have reported the utilization of these polysaccharides as drug carriers. *Bletilla striata* polysaccharides are water-soluble polysaccharides which lack a hydrophobic domain structure, so they are not good enough to coat fat-soluble drugs and the drug delivery yield is therefore low, making it necessary to find appropriate chemical means to modify them in order to improve their hydrogel strength and ability to coat drugs. Many polysaccharides, for instance pullulan [8] and carboxymethyl cellulose [9], had been successfully modified with cholesterol. This paper reports a cholesteryl succinate modification of a glucomannan, named BSP, isolated from *Bletilla striata* tubers in order to make it amphiphilic and self-assemble into nanoparticles in aqueous solution. This study will expand the application scope of BSP in drug delivery for an efficient use of the traditional Chinese drug and will also provide a new idea for the exploration of natural product resources.

## 2. Results and Discussion

### 2.1. Isolation and Purification of BSP

The crude polysaccharide (yield 16.9% of dried material) was obtained from the tubers of *Bletilla striata* via ultrasonic-assisted extraction, ethanol precipitation and Sevag reagent deproteination [10]. It was then purified using Sepharose CL-6B column chromatography to give the pure polysaccharide fraction BSP. BSP contained 96% total carbohydrate as determined by the phenol-sulfuric acid method [10], but did not contain uronic acid as determined by the *m*-hydroxydiphenyl method [11]. BSP had no absorption at 260 nm and 280 nm, showing it did not contain nucleic acid and protein. Sugar composition analysis revealed that BSP mainly consisted of mannose and glucose in the molar ratio of 3.5:1, consistent with the result that it contained no uronic acid. BSP exhibited a single and symmetrical peak both on GPC and HPGPC, showing that it was homogeneous. Its Mw of 20 kDa, as estimated by HPGPC using standard dextrans of known Mw, was lower than those of the polysaccharide fractions (135 kDa, 182 kDa and 235 kDa) extracted by hot water [4,5,12]. This might be due to the ultrasonic-assisted extraction used in this study resulting in some degradation of the water soluble polysaccharides.

### 2.2. Structural Analysis of BSP

**Figure 1:**
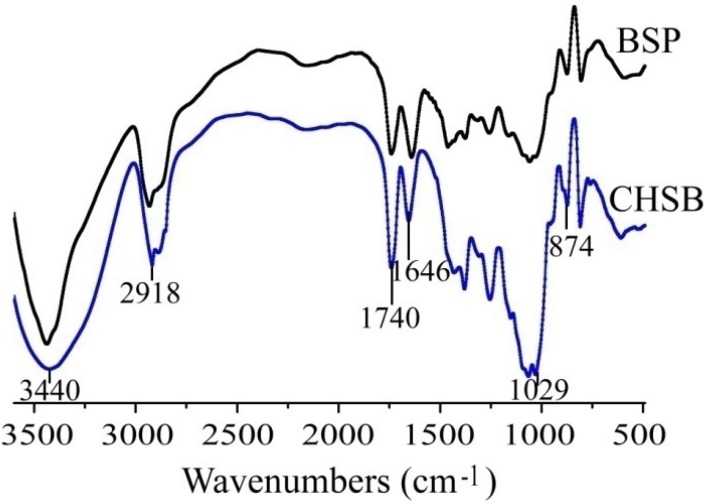
The FT-IR spectra of BSP and CHSB.

The FT-IR spectrum of BSP (Figure 1) showed the characteristic absorption of β-type glycosidic linkages at 874 cm^−1^ [6]. The absorption band at 3443 cm^−1^ was assigned to hydroxyl stretching vibrations, the band at 2925 cm^−1^ to C-H stretching vibrations, the band at 1743 cm^−1^ to the stretching vibration of the C=O in the ester carbonyl groups, and the band at 1646 cm^−1^ to bound water [13]. The absorption at 1029 cm^−1^ was due to the pyranose configuration of the residues.

The fully methylated BSP was hydrolyzed with acid, converted into alditol acetates and analyzed by GC-MS. The results, summarized in Table 1, suggest that BSP was mainly composed of (1→4)-linked-d-mannose and (1→4)-linked-d-glucose. The molar ratio of the two glycosidic linkages was in agreement with that of the monosaccharide composition in BSP.

**Table 1:** Methylation analysis data for BSP.

Methylated Sugar	Linkages Types	Molar Ratio (%)	Mass Fragment (*m/z*)
2, 3, 6-Me_3_-Man*p*	→4)-d-Man-(1→	76.4	43, 87, 101, 117
			129, 161, 203, 233, 277
2, 3, 6-Me_3_-Glc*p*	→4)-d-Glc-(1→	20.3	43, 87, 99, 101
			117, 129, 189, 233

**Figure 2:**
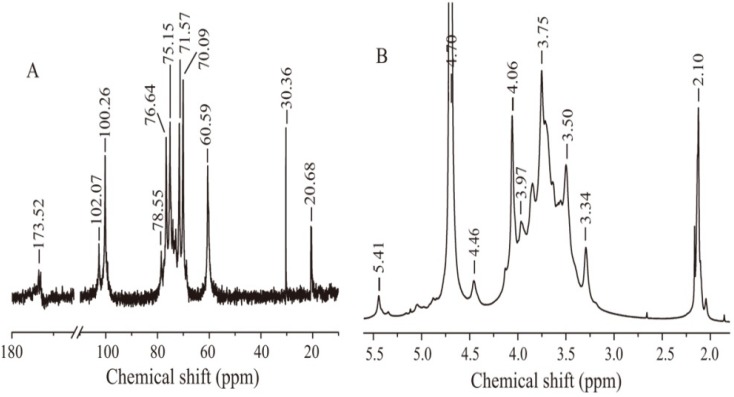
(**A**) ^13^C-NMR spectrum of BSP; (**B**) ^1^H-NMR spectrum of BSP.

**Figure 3:**
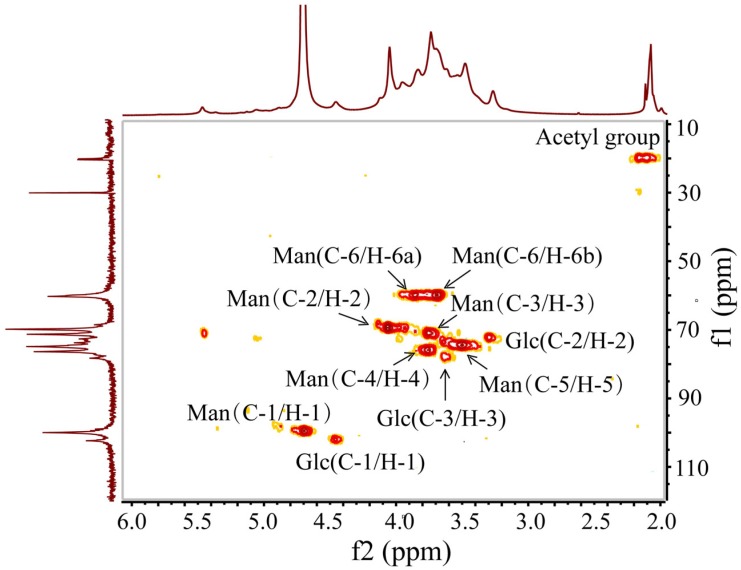
HSQC NMR spectrum of BSP.

Further structural features of BSP were determined by ^13^C- and ^1^H-NMR spectroscopy (Figure 2). Assignments of signals and identification of the sugar residues were done by combinations of two‑dimensional HSQC analysis (Figure 3) and comparison of the chemical shifts with published data [14,15,16]. All the results are summarized in Table 2. In the ^13^C-NMR spectrum, the signal at 30.4 ppm was due to the methyl carbon in the acetone used as the internal standard reference. The anomeric signal at 100.26 ppm was attributed to C-1 of 1,4-linked β-Man. Other signals at 70.09, 71.57, 76.64, 75.15 and 60.59 ppm were attributed to C-2, C-3, C-4, C-5 and C-6 of 1,4-linked β-Man, respectively. The anomeric signal at 102.07 ppm was assigned to 1,4-linked β-Glc, and the signal at 78.55 ppm belonged to the C-4 of 1,4-linked β-Glc. The signal at 173.5 ppm and 20.7 ppm were assigned to carbonyl carbon and methyl carbon in acetyl groups, respectively. This was consistent with the FT-IR result and suggested the presence of acetyl groups in BSP. 

In the ^1^H-NMR spectrum, signals at 4.70, 4.06, 3.72, 3.75, 3.50, 3.89/3.69 ppm were related to H-1 to H-6 of 1,4-linked β-Man, respectively. The signal of 4.46 ppm was attributed to H-1 of 1,4-linked β‑Glc. The highest-field signal, at 2.10 ppm, belonged to the methyl of an acetyl group. In the low field, there was a signal at 5.41 ppm, which might be assigned to H-2 of 2-*O*-acetyl-1,4-linked β-Man [17].

According to the methylation analysis, FT-IR and NMR results, BSP was thus determined to be an acetylglucomannan having a backbone of 1,4-linked β-d-Man and β-d-Glc in a ratio of ~3.5:1. This structure was different from the glucomannan (BSPb) fraction isolated from *Bletilla striata*, which was composed of 1,2-linked α-d-Man and 1,4-linked β-d-Glc with a ratio of 3:1 [6], but was similar to the polysaccharide “*Bletilla*-glucomannan” with both 1,4-linked β-d-Man and β-d-Glc in a ratio of 3:1 [4], and the polysaccharide BSPF2 with 1,4-linked β-d-Man and β-d-Glc in a ratio of 2:1, although three fifths of the Glc residues in BSPF2 also substituted by terminal Man residues [5]. The structural differences between BSP and other *Bletilla striata* polysaccharides might be caused by the different extraction methods. A polysaccharide with similar structure was found in the dried stem of *Dendrobium officinale*, which had a backbone of 1,4-linked β-d-Man and β-d-Glc, with branches at O-6 consisting of terminal and 1,3-linked Man, 1,3-linked Glc, and a small proportion of arabinofuranosyl residues at the terminal position [17]. An acetylated glucomannan was also obtained from *Cyrtopodium andersonii*, which had a backbone of 1,4-linked β-d-Man and β-d-Glc and substituted by acetyl groups at C-2 of the 1,4-linked β-d-Man units [18].

**Table 2:** ^13^C-NMR and ^1^H-NMR spectral assignments of BSP.

Sugar Residues	Chemical Shifts ^13^C/^1^H δ (ppm)					
	1	2	3	4	5	6
→4)-β-D-Man-(1→	100.26	70.09	71.57	76.64	75.15	60.59
	4.70	4.06	3.72	3.75	3.50	3.89/3.69
→4)-β-D-Glc-(1→	102.07	72.17	75.89	78.55	74.5	60.69
	4.46	3.34	3.76	3.64	3.58	3.77/3.65

### 2.3. Determination of Degree of Substitution (DS) for Cholesteryl Succinate Modified BSP (CHSB)

In order to increase the hydrophobic domain of BSP, it was modified by reaction with cholesteryl succinate (CHS) catalyzed by 1-ethyl-(3-dimethylaminopropyl) carbonylimine hydrochloride (EDC) and triethylamine. After CHS was connected to the BSP molecule, a hydrophobic modified BSP (CHSB) was obtained. The FT-IR spectra of BSP and CHSB are shown in Figure 1. Compared with the spectrum of BSP, the absorption at about 1740 cm^−1^, which was assigned to the stretching vibrations of the carbonyl group, was remarkably increased in CHSB with the substitution by CHS. At the same time, the increase of the methylene stretching vibration at about 2920 cm^−1^ was observed. These results indicated that the modification of BSP by CHS proceeded successfully.

It is reported that sulfuric acid could hydrolyze deoxycholate which was covalently bound to dextran-modified polysaccharides [19]. By detecting the amount of deoxycholate by UV spectrophotometry, the degree of substitution by deoxycholate in the modified polysaccharide could be obtained. Inspired by this method, ammonia ferric sulfate was used in this study to hydrolyze CHSB and the degree of substitution by CHS in CHSB was determined by a colorimetric method [20]. Using Equation (1), the degree of substitution for CHSB was determined to be 3.2%:

DS = (C/M_1_)/((m − C)/M_2_) (1)

where, M_1_ is the molar mass of cholesterol 387; M_2_ is the molar mass of a sugar unit structure 162; C is for the concentration of cholesterol and m is the weight of CHSB 15 mg.

### 2.4. Determination of the Critical Micelle Concentration (CMC) of CHSB by Pyrene Fluorescence Probe Spectrometry

Pyrene is used as a hydrophobic fluorescent probe [21] due to its low solubility, self-quenching effect and relatively low fluorescence intensity in polar environment. Therefore, when a polar solvent (water) exists in a micellar or hydrophobic domain, pyrene will spontaneously metastasize to the nonpolar environment, leading to significant increase of fluorescence intensity. In aqueous solution, CHSB could form a hydrophobic domain, therefore pyrene would shift into this hydrophobic domain, producing changes in the emission spectrum accordingly. By measuring the fluorescence intensity at I_372_ and I_383_, the CMC of CHSB could be determined.

**Figure 4:**
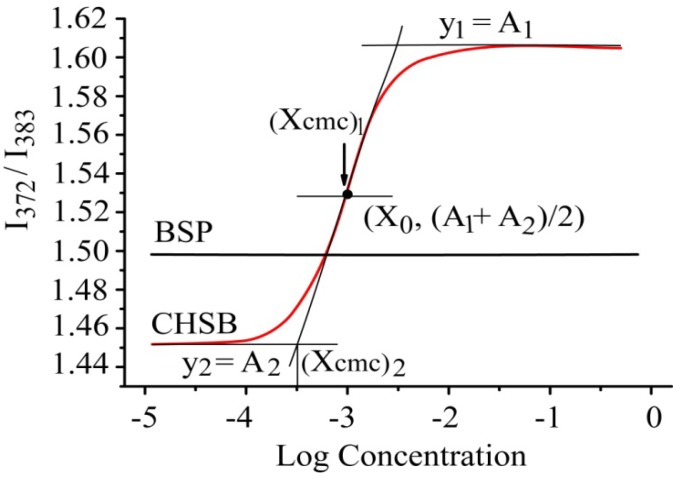
The diagram of the fluorescence excitation spectra intensity ratio I_372_/I_383_ and the logarithmic (Log) concentration of CHSB and BSP. BSP was used as a control.

Figure 4 showed the fluorescence excitation ratio of I_372_/I_383_ for CHSB and BSP in correlation with log concentration. The concentration of BSP had nothing to do with the ratio of I_372_/I_383_. When the concentration of CHSB was low, the ratio of I_372_/I_383_ basically remained unchanged. When the concentration exceeded the critical concentration, the ratio of I_372_/I_383_ increased dramatically along with the increase of concentration. Aguiar *et al.* [22] reported that the relationship between the fluorescence excitation ratio I_372_/I_383_ and the concentration was in accordance with the Boltzamann curve rule:

y = [(A_1_ − A_2_)/(1 + e^(x − x_0_)/Δx^] + A_2_ (2)

In Equation (2), x_0_ is the center of the sigmoid and x is directly related to the independent variable range where the abrupt change of the dependent variable occurs. A_1_ is the ratio of I_372_/I_383_ under surfactant with low concentration and A_2_ is the ratio of I_372_/I_383_ under surfactant with high concentration. When x_0_/Δx < 10 (where Δx = ((x_CMC_)_2_ − x_0_)/2), CMC = (x_CMC_)_1_; otherwise, CMC = (x_CMC_)_2_. After nonlinear curve fitting, the following results were obtained: (x_CMC_)_1_ = 0.001 mg/mL (x_CMC_)_2_ = 0.483 mg/mL, x_0_ = 9.6 × 10^−4^ mg/mL. Therefore, x_0_/Δx = 0.004 < 10. According to what is mentioned above, CMC = (x_CMC_)_1_, which meant the intersection point corresponded to the CMC. This result showed that the CMC of CHSB was 0.001 mg/mL, less than that of many other small molecule surfactants, for example, the CMC of sodium dodecyl sulfate was 2.3 mg/mL [23] and the CMC of deoxycholate was 1.0 mg/mL [24]. Therefore, CHSB was a novel kind of amphiphilic macromolecule material and could self-assemble into nanoparticles in aqueous solution. This low CMC of CHSB shows that it would have a good stability in physiological liquid environment and is expected to be useful in drug delivery systems. Cholesteryl groups are hydrophobic segments which possess good biocompatibility which is a great ability to drive self-assembly, as well as strong potential interactions with cholesteryl receptors on the cell surface. Cholesteryl-modified hydroxypropyl cellulose had been well studied [25,26]. These cellulose derivatives had good loading capacity for hydrophobic drugs and controlled-release properties [27]. Therefore, we hypothesize that CHSB would be a novel nanocarrier for hydrophobic drug delivery.

## 3. Experimental Section

### 3.1. Materials

The tubers of *Bletilla striata* were bought from Nanjing Institute of Comprehensive Utilization of Wild Plant. Sepharose CL-6B gel was obtained from Amersham Pharmacia Biotech (Uppsala, Sweden). All other reagents used were of analytical grade made in China.

### 3.2. General Methods

The total sugar content was determined by a phenol-sulfuric acid assay using Glc as standard [28]. Sepharose CL-6B chromatography was performed on a 1.5 × 90 cm column, eluating with 0.15 M NaCl at a flow rate of 0.15 mL/min. Dialysis was carried out using tubing with Mw cut-off 3500 Da (for globular proteins). FT-IR spectra were obtained in a range of 4000–400 cm^−1^ on a Nicolet 6700 FT-IR spectrometer (ThermoScientific, Waltham, MA, USA) with a DTGS detector. The sample was measured as a film on KBr discs. The NMR experiments were conducted as described by Zhang [10]. BSP (20 mg) was dissolved in D_2_O (99.8%, 0.5 mL), freeze-dried, re-dissolved in D_2_O (0.5 mL), and centrifuged to remove the excess sample.

### 3.3. Isolation and Purification

*Bletilla striata* tuber powder (20 g) was immersed in 20 volumes of distilled water and submitted to ultrasonic-assisted extraction with water at 80 °C for 10 min. The aqueous filtrates were combined and concentrated to one-tenth of the original volume. To precipitate the polysaccharides, 95% ethanol was added to the aqueous filtrates to reach 80% of the final volume. The polysaccharides were collected by centrifugation and dried in vacuum. The supernatant was treated with Sevag reagent (1:4 n-butanol/chloroform, v/v) to remove the free proteins [10]. The deproteinated polysaccharide (5 g) was dissolved in distilled water (10 mg/mL) and centrifuged. The supernatant was applied on a Sepharose CL-6B column (3.0 × 90 cm) and eluted with 0.15 M NaCl solution at a flow rate of 0.5 mL/min. The eluate was collected 10 mL per tube. The main peak was collected according to the sugar profile detected by phenol-sulfuric acid method, concentrated, dialyzed and lyophilized to yield BSP.

### 3.4. Homogeneity and Molecular Weight

Homogeneity and molecular weight were determined by HPGPC performed on a Shimadzu, 10Avp linked gel filtration column of TSK-G4000 PW_XL_, eluting with 0.2 M NaCl at 0.5 mL/min, 35.0 ± 0.1 °C, monitored using a refractive index RID-10A detector (Shimadzu, Kyoto, Japan). A sample solution 20 µL (5 mg/mL) was injected in the column. The gel filtration column was calibrated by standard dextrans (410 kDa, 150 kDa, 50 kDa, 5 kDa) using linear regression.

### 3.5. Monosaccharide Composition

Monosaccharide analysis was performed by a HPLC method as described by Zhang [10]. The polysaccharide BSP (2 mg) was hydrolyzed using 1.0 mL anhydrous methanol containing 2 M HCl at 80 °C for 16 h and then with 1.0 mL of 2 M CF_3_COOH (0.5 mL) at 120 °C for 1 h. The hydrolysis-product was derivatized with 0.5 M 1-phenyl-3-methyl-5-pyrazolone derivatives and 0.3 M NaOH. After neutralization with 0.3 M HCl, the derivatives were analyzed by HPLC on a DIKMA Inertsil ODS-3 column (150 × 4.6 mm i.d.) with a guard column on a Shimadzu HPLC system (LC-10ATvp pump and UV-VIS detector) and monitored by UV absorbance at 245 nm.

### 3.6. Methylation Analysis

BSP was methylated using the Ciucanu and Kerek method [29]. BSP (20 mg) was dissolved in DMSO (2 mL) and methylated by treatment with NaOH-DMSO (2 mL) suspension and iodomethane (1.8 mL).The reaction mixture was extracted with CHCl_3_, and then the solvent was removed by vacuum evaporation. Complete methylation was confirmed by the disappearance of the -OH band (3200–3700 cm^−1^) in the FT-IR spectrum. The permethylated product was hydrolyzed subsequently by HCOOH (85%, 0.5 mL) for 4 h at 100 °C, and then by CF_3_COOH (2 M, 1 mL) for 6 h at 100 °C. The mixture was evaporated to dryness, followed by reduction with NaBH_4_ and acetylation with acetic anhydride. The resulting mixture of the methylated alditol acetates were analyzed by GC-MS using a Shimadzu GC-14C instrument.

### 3.7. Synthesis of Cholesteryl Succinate

Cholesterol (25 g) and succinic anhydride (20 g) were dissolved in anhydrous pyridine (20 mL) and maintained at room temperature for 48 h. The pH was adjusted to 1–2 with hydrochloric acid solution under ice cooling (hydrochloric acid/ice/water = 12/50/40). The mixture was kept at 4 °C overnight. After filtration, the precipitate was collected and washed with distilled water until pH > 5. The precipitate was recrystallized in ethyl acetate/ethanol system and dried at 80 °C to obtain a pure white needle-like cholesteryl succinate product.

### 3.8. Synthesis Cholesteryl Succinate - BSP (CHSB)

BSP polysaccharide (0.5 g) was dissolved in dimethyl formamide (DMF, 15 mL). CHS, EDC and triethylamine (EDC/CHS/triethylamine = 1.2/1/1) were dissolved in DMF (10 mL) and stirred at room temperature for 1 h. The above solution was added to the BSP polysaccharide solution and kept for 48 h. Anhydrous ethanol (200 mL) was added to the reaction mixture and a white precipitate was obtained. The resulting precipitate was separated by centrifugation, washed subsequently with ethanol, tetrahydrofuran and ether and finally dried at 80 °C.

### 3.9. Determination of the Degree of Substitution of CHSB

CHSB (7.5 mg) was mixed with 50% KOH solution (4.0 mL) and anhydrous ethanol (2.0 mL) in a 25 mL colorimetric tube. The mixture was submitted to ultrasonic-assisted dissolution for 5 min and saponification for 2 h at 65 °C on a water bath under shaking. After complete saponification, 3 mL 5% NaCl solution was added to the mixed solution and cooled at room temperature. After addition of petroleum ether (10 mL) with vigorous stirring for 1 min, it was left for stratification. One mL of the upper petroleum ether solution was drawn into a stoppered colorimetric tube and was dried under natural evaporation at 65 °C water bath. Ethanol (1 mL) was added and jiggled to make the cholesterol dissolve. The degree of substitution for CHSB was calculated from the change of absorption at 560 nm using the standard curve of cholesterol and the formula DS = (C/M_1_)/((m − C)/M_2_), where M_1_ is the molar mass of cholesterol 387; M_2_ is the molar mass of a sugar unit structure 162; C is for the concentration of cholesterol and m is the weight of CHSB 15 mg.

### 3.10. Determination of CMC of CHSB

For steady-state pyrene fluorescence probe spectrometry, the CHSB sample was dissolved in 2% acetic acid solution (1 mg/mL) and dialyzed against distilled water for 24 h. The non-dialyzed portion was filtered with a microporous membrane and the filtrate was submitted to ultrasonic-assisted treatment at 37 °C [30] followed by addition of methanol-pyrene solution in a 10 mL flask. The concentration of pyrene was adjusted to 3 × 10^−6^ mol/L after adding CHSB. The solution was submitted to ultrasonic-assisted treatment for 40 min and put on 37 °C water bath for 1 h under oscillation. After scanning at wavenumbers from 350 nm to 450 nm, the fluorescence emission spectrum was obtained and the excitation wavelength was set at 335 nm. By recording the fluorescence intensity at the absorption of 372 nm and 383 nm [31], the ratio of I_372_/I_383_ to the numerical log concentration was plotted to obtain CMC of CHSB, which corresponded to the middle point of curve inflexion.

## 4. Conclusions

From the results obtained in our study, we could conclude that the polysaccharide BSP extracted from *Bletilla striata* was a glucomannan with a Mw of 20 kDa. Methylation analysis, FT-IR and NMR spectra revealed that BSP was an acetylglucomannan having a backbone of 1,4-linked β-d-Man and β-d-Glc in a ratio of ~3.5:1. A chemical method for the modification of glucomannan by cholesteryl succinate was also established in this study and a modified product CHSB was obtained. The degree of substitution by cholesteryl succinate in CHSB was 3.2%. Using the steady-state fluorescence probe method, the critical micelle concentration of CHSB was determined to be 0.001 mg/mL, less than that of many other small molecule surfactants, which indicated CHSB was a novel kind of amphiphilic macromolecule material and could self-assemble into nanoparticles in aqueous solution. This study provided data and information that should help expand the utilization of BSP in drug delivery studies, expand the traditional Chinese drug industry and provide a novel concept for the exploration of natural product resources.
